# Supplementary material for: Bayesian refinement of protein structures and ensembles against SAXS data using molecular dynamics
Source: PLoS Comput Biol. 2017 Oct 18;13(10):e1005800. doi: 10.1371/journal.pcbi.1005800 (PMC5662244; doi:10.1371/journal.pcbi.1005800)
Supplement: S1 Archive — (BZ2) [file pcbi.1005800.s011.tar.bz2 › gromacs_4.62_bayesian_saxs_refi/share/html/online/g_densmap.html]

g\_densmap


| g\_densmap Main Table of Contents |

**VERSION 4.6  
Sat 19 Jan 2013**

---

### Description

g\_densmap computes 2D number-density maps.
It can make planar and axial-radial density maps.
The output .xpm file can be visualized with for instance xv
and can be converted to postscript with xpm2ps.
Optionally, output can be in text form to a .dat file with -od, instead of the usual .xpm file with -o.

The default analysis is a 2-D number-density map for a selected
group of atoms in the x-y plane.
The averaging direction can be changed with the option -aver.
When -xmin and/or -xmax are set only atoms that are
within the limit(s) in the averaging direction are taken into account.
The grid spacing is set with the option -bin.
When -n1 or -n2 is non-zero, the grid
size is set by this option.
Box size fluctuations are properly taken into account.

When options -amax and -rmax are set, an axial-radial
number-density map is made. Three groups should be supplied, the centers
of mass of the first two groups define the axis, the third defines the
analysis group. The axial direction goes from -amax to +amax, where
the center is defined as the midpoint between the centers of mass and
the positive direction goes from the first to the second center of mass.
The radial direction goes from 0 to rmax or from -rmax to +rmax
when the -mirror option has been set.

The normalization of the output is set with the -unit option.
The default produces a true number density. Unit nm-2 leaves out
the normalization for the averaging or the angular direction.
Option count produces the count for each grid cell.
When you do not want the scale in the output to go
from zero to the maximum density, you can set the maximum
with the option -dmax.

### Files

| option | filename | type | description |
| --- | --- | --- | --- |
| **-f** | traj.xtc | Input | Trajectory: xtc trr trj gro g96 pdb cpt |
| **-s** | topol.tpr | Input, Opt. | Structure+mass(db): tpr tpb tpa gro g96 pdb |
| **-n** | index.ndx | Input, Opt. | Index file |
| **-od** | densmap.dat | Output, Opt. | Generic data file |
| **-o** | densmap.xpm | Output | X PixMap compatible matrix file |

### Other options

| option | type | default | description |
| --- | --- | --- | --- |
| **-[no]h** | bool | no | Print help info and quit || **-[no]version** | bool | no | Print version info and quit || **-nice** | int | 19 | Set the nicelevel || **-b** | time | 0 | First frame (ps) to read from trajectory || **-e** | time | 0 | Last frame (ps) to read from trajectory || **-dt** | time | 0 | Only use frame when t MOD dt = first time (ps) || **-[no]w** | bool | no | View output .xvg, .xpm, .eps and .pdb files || **-bin** | real | 0.02 | Grid size (nm) || **-aver** | enum | z | The direction to average over: z, y or x || **-xmin** | real | -1 | Minimum coordinate for averaging || **-xmax** | real | -1 | Maximum coordinate for averaging || **-n1** | int | 0 | Number of grid cells in the first direction || **-n2** | int | 0 | Number of grid cells in the second direction || **-amax** | real | 0 | Maximum axial distance from the center || **-rmax** | real | 0 | Maximum radial distance || **-[no]mirror** | bool | no | Add the mirror image below the axial axis || **-[no]sums** | bool | no | Print density sums (1D map) to stdout || **-unit** | enum | nm-3 | Unit for the output: nm-3, nm-2 or count || **-dmin** | real | 0 | Minimum density in output || **-dmax** | real | 0 | Maximum density in output (0 means calculate it) |

---

http://www.gromacs.org  
gromacs@gromacs.org
